# Hypocretin (orexin) regulation of sleep-to-wake transitions

**DOI:** 10.3389/fphar.2014.00016

**Published:** 2014-02-12

**Authors:** Luis de Lecea, Ramón Huerta

**Affiliations:** ^1^Department of Psychiatry and Behavioral Sciences, Stanford University School of Medicine, Stanford, CA, USA; ^2^BioCircuits Institute, University of California, San Diego, La Jolla, CA, USA

**Keywords:** hypothalamus, orexin, sleep, optogenetics, hypervigilance/avoidance

## Abstract

The hypocretin (Hcrt), also known as orexin, peptides are essential for arousal stability. Here we discuss background information about the interaction of Hcrt with other neuromodulators, including norepinephrine and acetylcholine probed with optogenetics. We conclude that Hcrt neurons integrate metabolic, circadian and limbic inputs and convey this information to a network of neuromodulators, each of which has a different role on the dynamic of sleep-to-wake transitions. This model may prove useful to predict the effects of orexin receptor antagonists in sleep disorders and other conditions.

## INTRODUCTION

Transitions between states of vigilance have long been associated with changes in cortical excitability associated with changes in the activity of monoamines and neuromodulators (Steriade, 2003). Steriade and McCarley (1990), Steriade et al. (1993), Steriade (2003) performed intracellular recordings of cortical neurons in different brain states and proposed that the concerted activity of norepinephrine, histamine, acetylcholine, and glutamate was sufficient to induce a sleep-to-wake transition. However, the mechanisms underlying the precise coordination of sleep states have remained poorly understood. The discovery of the hypocretins (Hcrts), also known as orexins, has provided a missing link in the regulation of states of vigilance.

### THE HYPOCRETINS/OREXINS: CRITICAL REGULATORS OF AROUSAL STABILITY

Soon after their discovery in 1998 (de Lecea et al., 1998; Sakurai et al., 1998), two groups described the association between Hcrt deficiency and the sleep disorder narcolepsy (Chemelli et al., 1999; Lin et al., 1999; Nishino et al., 2000, 2001; Peyron et al., 2000; Thannickal et al., 2000). Several studies have shown that the Hcrt knockout (KO) or Hcrt-R2 deficient (Mochizuki et al., 2011) mice have normal amounts of sleep and wakefulness across the light/dark cycle (Mochizuki et al., 2004) but exhibit an increased instability of behavior states. Dogs with mutations in Hcrt R2 exhibit narcolepsy with cataplexy (Lin et al., 1999). Patients that suffer from narcolepsy with cataplexy have very low levels of Hcrt-1 in their CSF (Nishino et al., 2000; Peyron et al., 2000; Thannickal et al., 2000). These deficits are likely caused by selective degeneration of Hcrt cells (rather than down regulation of the Hcrt gene) because other markers that colocalize with Hcrt are also reduced in narcoleptic patients (Crocker et al., 2005). Indeed, a recent study has revealed epitopes in the Hcrt precursor sequence that trigger activation of CD4 T-cells (De la Herran-Arita et al., 2013). All of these data clearly demonstrate that Hcrt signaling is necessary for arousal stability.

The first recordings of Hcrt neurons *in vitro* indicated that these cells are spontaneously active and responsive to multiple stimuli. Studies by Fujiki et al. (2001) using microdialysis and Estabrooke et al. (2001) using c-fos mapping revealed a circadian modulation of Hcrt peptide concentration in brain tissue. Parallel studies using juxtacellular recordings in head-fixed or freely moving animals showed that, surprisingly, Hcrt activity is mostly phasic, and precedes sleep-to-wake transitions by 10–20 s (Lee et al., 2005; Mileykovskiy et al., 2005). The question remained as to whether this phasic activity of Hcrt neurons was permissive or instructive for awakenings. In the first *in vivo* application of optogenetics in behaving animals, Adamantidis et al. (2007) found the photostimulation-induced activation of Hcrt neurons specifically increases the probability of transitions from sleep to wake (Adamantidis et al., 2007). This induction was frequency-dependent as only frequencies > 5Hz increased awakening probability. Semi-chronic stimulation of Hcrt neurons did not result in significant increases in the amount of non-rapid eye movement (NREM) sleep suggesting that phasic activation of Hcrt cells is involved in the transition to wake, but not in wake maintenance. Optogenetic silencing of Hcrt neurons induces sleep during the light phase, but not during the dark phase (Tsunematsu et al., 2011). These findings were further validated using a newly developed pharmacogenetic technology designer receptors exclusively activated by designer drugs (DREADDs; Sasaki et al., 2011) that allows the modulation of neural activity with temporal resolution of several hours. Therefore, the Hcrt system acts as a regulator of behavior states by modulating the arousal threshold (Sutcliffe and de Lecea, 2002), so that the organism can keep appropriate and adequate wakefulness to cope with fluctuations of the external and internal environments.

Then, does the existence of two subtypes of receptors account for these two aspects of functions of Hcrt? Hcrt-R2 deficient mice display fragmented wakefulness similar to the narcoleptic phenotype whereas Hcrt-R1-knockout mice only show a mild sleep disorder (Willie et al., 2001; Mieda et al., 2011). However, the double Hcrt-R1 and Hcrt-R2 receptor knockout mice suffer a more severe deficit in sleep–wake cycle than Hcrt-R2-knockouts, which exhibit a low degree of cataplexy and rapid eye movement sleep (REM) sleep intrusion (Chemelli et al., 1999; Willie et al., 2003; Mieda et al., 2011). Therefore, both the Hcrt-R1 and Hcrt-R2 are essential in the process of keeping a stable sleep/wakefulness cycle, with a larger contribution of Hcrt-R2. On the other hand, a recent study revealed that the Hcrt-1-mediated promotion of wakefulness was attenuated in both Hcrt-R1 and Hcrt-R2-knockout mice, and both receptors seem to be associated with the suppression of REM sleep (Mieda et al., 2011). However, a recently functional magnetic resonance imaging (fMRI) study revealed that the antagonist of Hcrt-R2 but not Hcrt-R1 increased REM, non-REM and total sleep-time, suggesting the distinct roles of the two receptors (Gozzi et al., 2011). Also, the recent development of Hcrt receptor selective antagonists showed that Hcrtr-1 blockade attenuates Hcrt-R2 antagonism and revealed complex interactions between Hcrt-R1 and Hcrt-R2 (Dugovic et al., 2009). Selective and non-selective Hcrt receptor antagonists have recently completed Phase III clinical trials for the treatment of insomnia (Herring et al., 2012), a remarkable development from a gene product discovered only 15 years ago.

### AFFERENTS TO HCRT NEURONS

Anatomical and electrophysiological evidence accumulated over the last decade has shown that at least 10 other transmitters and hormone are sensed by Hcrt cells (Inutsuka and Yamanaka, 2013).* Most notably, NE, 5HT, NPY, CCK, ghrelin, nicotinic, and muscarinic acetylcholine, AMPA, NMDA Glutamate, GABAa, and GABAb receptors are expressed by Hcrt cells *(Sakurai, 2007). In the absence of co-localization studies, it is assumed that most of these receptors are randomly distributed within the Hcrt population.* Thus, as a network, Hcrt neurons receive information about the general excitability and arousal (Glu, GABA, ACh, NE, 5HT), feeding and metabolic state (NPY, Ghrelin, Leptin, and CCK). *Interestingly, Hcrt neurons may change their sensitivity to NE after sleep deprivation (Grivel et al., 2005), thus providing a mechanism through which Hcrt cells sense previous sleep history and homeostatic balance. Anatomical afferents have revealed several key areas that send axons to Hcrt cells (Sakurai et al., 2005; Yoshida et al., 2006) including the bed nucleus of the stria terminalis, the amygdala, and the medial septum, supporting a role of the limbic system in regulating Hcrt responses.

### EFFECTORS OF HCRT NEURONS: THE MONOAMINES

The flip/flop model of sleep–wake cycle (Saper et al., 2010) posits that monoamines stimulate neocortical neurons and inhibit sleep centers to promote wakefulness. Importantly, these monoaminergic neurons in tuberomammillary nucleus (TMN, Histaminergic), locus coeruleus (LC, noradrenergic), dorsal raphe nuclei (DRN, serotoninergic), ventral periaqueductal gray matter (vPAG, dopaminergic) receive dense projections of Hcrt neurons (Peyron et al., 1998; Saper et al., 2005), consist with the distribution of HcrtRs (Marcus et al., 2001). LC neurons mainly express Hcrt-R1, TMN neurons mostly Hcrt-R2 whereas DRN express both Hcrt-R1 and Hcrt-R2. Moreover, Hcrt neurons exhibit parallel firing patterns with monoaminergic neurons that represent tonic firing during wakefulness especially during active wakefulness, mild firing during slow wave sleep, and then silent during REM sleep (Estabrooke et al., 2001; Lee et al., 2005; Mileykovskiy et al., 2005), except its intensive firing at the transition to wakefulness. These data are also consistent with the oscillation of extracellular Hcrt-1 concentration that peak during the waking state and fall down to about half their max levels during sleep (Yoshida et al., 2001; Zeitzer et al., 2003). These observations suggest that Hcrt system stabilizes wakefulness through driving the arousal system during the arousal state (Saper et al., 2010).

Indeed, *in vitro* electrophysiological studies showed that Hcrt activates the TMN histaminergic (Bayer et al., 2001; Eriksson et al., 2001; Huang et al., 2001; Schone et al., 2012), LC noradrenergic (Hagan et al., 1999) and DRN serotoninergic (Liu et al., 2002) neurons, and *in vivo* experiments revealed the involvement of LC and the Hcrt-R1 in LC (Bourgin et al., 2000), as well as the histamine 1R (H1R; Huang et al., 2001) and the Hcrt-R2 signaling in TMN (Mochizuki et al., 2011) in Hcrt-induced arousal (Schone et al., 2012). However, recent reports found that Hcrt-mediated sleep-to-wake transition in mice did not depend on the histaminergic system (Carter et al., 2009a) and the mice could display a normal sleep/wake pattern in the condition that both H1R and Hcrt-R1 are deficient (Hondo et al., 2010). The role of Histaminergic cells may be more related to maintenance of the awake state, as histamine-deficient HDC knockout mice only show decreased arousal in new environments

Moreover, Lu and Greco (2006) demonstrated that loss of dopaminergic neurons in vPAG, a rostral extension of the ventral tegmental area (VTA), results in a reduction of wakefulness by 20% accompanied by increase of NREM, REM sleep. This finding is supported by a recent report (Kaur et al., 2009) that identified the Hcrt -vPAG circuit, whose activity suppresses REM sleep but not non- REM sleep. On the other hand, Hcrt neurons receive inhibition innervation from noradrenergic (Li et al., 2002), serotoninergic (Yamanaka et al., 2003; Kumar et al., 2007) and dopaminergic (Yamanaka et al., 2006) inputs whereas the histamine has little, if any, effect (Yamanaka et al., 2003). The role of noradrenergic innervation to Hcrt cells remains controversial, as some reports show excitatory effects in rats and others demonstrate inhibitory action (Grivel et al., 2005).

Cholinergic neurons in pedunculopontine tegmental nucleus/laterodorsal tegmental nucleus (PPT/LDT) fire most rapidly during wakefulness and REM sleep but slowly during NREM sleep (Saper et al., 2005), suggesting that they help to maintain the cortical activation in the states of wakefulness and REM sleep. Application of Hcrt-1 into LDT results in a significant increase of wakefulness but a decrease of amount rather than the duration of REM sleep (Xi et al., 2001). *In vitro* studies have shown that carbachol, a cholinergic agonist, excites Hcrt neurons (Bayer et al., 2005). In addition, intracerebroventricular (ICV) administration of Hcrt -1 (Piper et al., 2000) or local application into the LC (Bourgin et al., 2000) basal forebrain (Espana et al., 2001; Thakkar et al., 2001), lateral preoptic area (Methippara et al., 2000) increases the waking time at the expense of sleep. In summary, Hcrt-induced arousal is modulated not only by monoaminergic neurons, but also needs the participation of cholinergic neurons in the PPT/LDT and basal forebrain.

Importantly, the Hcrt system may be modulated by the circadian clock and homeostatic states (Deboer et al., 2004; Carter et al., 2009b; Appelbaum et al., 2010). Even though there is no evidence of a direct synaptic connection between the Suprachiasmatic nucleus (SCN) and Hcrt cells, the circadian clock drives Hcrt system through the output circuits of the Suprachiasmatic nucleus (SCN) (Deurveilher and Semba, 2005). *The internal clock molecular machinery in Hcrt neurons (i.e., per, CLOCK, BMAL1, etc.) may also influence neuronal excitability during the light/dark cycle, effectively integrating circadian cues without direct Suprachiasmatic nucleus (SCN) connectivity.* Additionally, local modulation of Hcrt neurons by Hcrt release (Li et al., 2002; Yamanaka et al., 2010), melanin-concentrating hormone (MCH; Rao et al., 2008; Hassani et al., 2009) or LepRB neurons (Leinninger et al., 2011) may also be important in the circadian stabilization of proper sleep–wake cycle. Intrinsic plasticity mechanisms may regulate the firing probability of Hcrt cells during day and night (Appelbaum et al., 2010). During the wakefulness period, tonic excitation of Hcrt neurons may be enhanced when the organism faces certain stressors like emotional stimulation, which involves the limbic input (Tsujino and Sakurai, 2009). Horvath and Gao (2005)* proposed that plasticity mechanisms in Hcrt cells are critical players in the connection between arousal, metabolism, and brain reward function.* Adamantidis and de Lecea (2008a,b) have suggested that Hcrt exerts different functions on different timescales: phasic activity lasting 1–10 s that would be mostly responsible for the state transitions, and a clock-regulated oscillation that would encode superimposed information about metabolic and circadian state.

### TRANSLATIONAL CONSIDERATIONS

The Hcrt system has been involved in a myriad of pathological processes, including Parkinson’s (PD; Drouot et al., 2003; Asai et al., 2008; Baumann et al., 2008; Fronczek et al., 2008), Alzheimer’s (AD; Kang et al., 2009; Scammell et al., 2012), anxiety and panic disorders (Johnson et al., 2010) and depression (Salomon et al., 2003; Borgland and Labouebe, 2010). The mechanisms of these associations vary broadly, particularly in the neurodegenerative diseases. For instance, some studies have shown that a Hcrt receptor antagonist can reduce plaque formation in animal models of AD. However, other reports have shown the same prevalence of AD in narcoleptics and control patients. The role of Hcrt in panic and anxiety may be mediated through several of its connections to the paraventricular hypothalamus and brainstem nuclei. Similarly, the projections of Hcrt cells to serotonergic dorsal raphe neurons and periaqueductal gray suggest a possible mechanism of modulation of 5HT release and mood. Hcrt R1 knockout animals and pharmacological inhibition reduces time of immobility in the tail suspension test (Scott et al., 2011). In contrast, Hcrt r2 knockout animals showed increased despair. Future development of Hcrtr1 selective antagonists may thus proof useful in the treatment of depression.

### OUTPUT OF HCRT NEURONS

**FIGURE 1:**
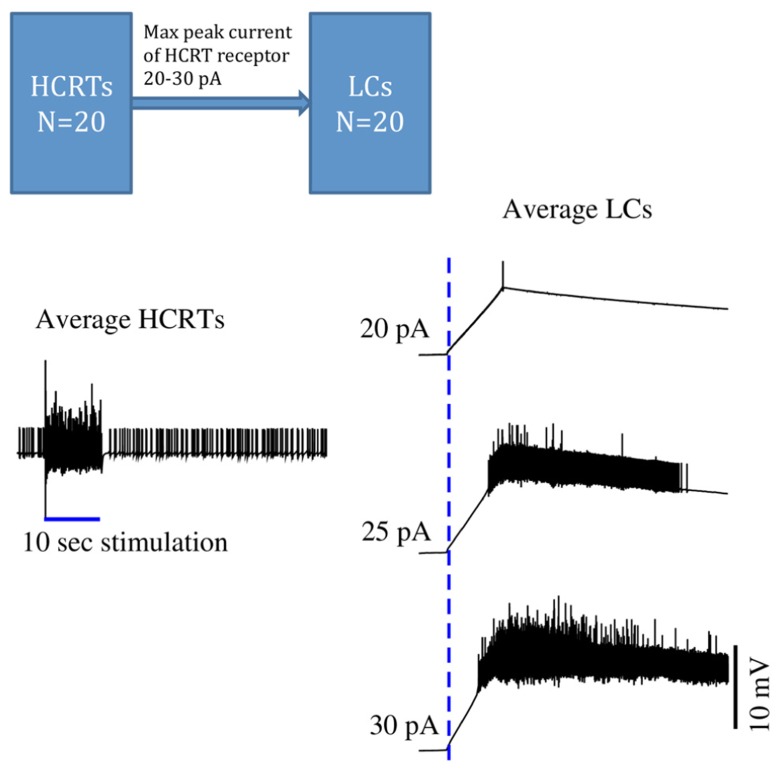
**Time series of in silico conductance-based models of Hcrt and LC neurons.** During sleep, both Hcrt and LC neurons are relatively quiescent. Once Hcrt neurons have integrated all of their inputs, including metabolic, circadian, and limbic states, they initiate a train of spikes (here mimicked by a virtual stimulation) that release glutamate and eventually Hcrt on post-synaptic neurons. *This model is made of 40 neurons using the same conductance-based model published in *(Carter et al., 2012).* Excitability of Hcrt and LC neurons in this model was modified by using the V*_t _value -52 mV and is regulated by randomly selecting the V_t _values centered at -52.0 mV using a Gaussian process with standard deviation of 1 mV. HCRT neurons are stimulated during 10 *s with a 5 pA current as indicated by a blue straight line on the left hand side.* Glutamate release elicits a slow depolarization on LC neurons, and cumulative release of Hcrt reaches a threshold that results in a train of spikes of LC neurons. *Three maximal currents elicited by HCRT receptors into the LCs are used: 20, 25, and 30 pA. The delayed excitability of LC neurons is very sensitive by only modifying the peak current by 10%. The dotted blue line indicates when the HCRTs start to be stimulated. This model is a simplification because it ignores the effect of regulatory inhibitory neurons widely present in hypothalamic circuits. Further work should show the stabilization of the LCs by using GABAergic circuits.* Carter et al. (2010) demonstrated that subtle stimulation of LC neurons, reaching 20 pulses in 5 s, deterministically results in an awakening.

Peyron et al. (1998) described a broad distribution of Hcrt fibers throughout the brain. Very few Hcrt projections have been studied in detail. The LC receives a very dense network of Hcrt-immunopositive axon terminals, and the connectivity between Hcrt and LC neurons has been shown to be monosynaptic. *Recently, *Carter et al. (2012)* have suggested a conductance-based computational model by which a short (> 10 s) period of phasic Hcrt activity enhances the excitability of post-synaptic LC neurons through conductances that elevate the concentration of intracellular calcium* (**Figure 1**). *Hcrt action on post-synaptic targets is remarkably slow* (Burlet et al., 2002; Kohlmeier et al., 2008)*, lasting several seconds, a dynamic that is consistent with the wake latencies observed after optogenetic stimulation of Hcrt cells in vivo* (Mileykovskiy et al., 2005)*. Release of Hcrt, either synaptic or extrasynaptic, increases the excitability of LC neurons.* Since optogenetic studies have showed that only a few light pulses (~20) to LC neurons are sufficient to induce behavioral sleep-to-wake transitions, mild excitation of LC neurons by other afferents within ~10 s of Hcrt-enhanced excitability would reach the threshold of an awakening with high probability (**Figure 1**).

**FIGURE 2:**
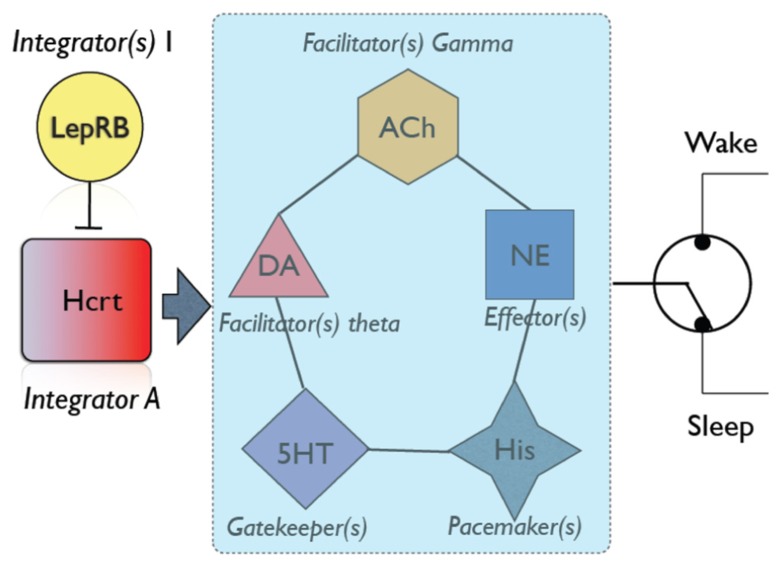
**An overall schematic of neuromodulators involved in sleep/wake transitions.** Hcrt neurons play a central role in integrating information from metabolic state [as demonstrated by numerous authors, see Yamanaka et al. (2003)], stress (Winsky-Sommerer et al., 2004) and circadian factors. Additional neuronal groups may be involved in integrating other physiological variables [e.g., LepRB neurons; (Louis et al., 2010)]. If physiological variables favor sleep (i.e., appropriate circadian time, strong sleep pressure, low energy demands), Hcrt neurons are silent, and this would be interpreted by cortical circuits as a signal of sleep maintenance (Morairty et al., 2013). Otherwise, Hcrt neurons send information to a network of arousal systems, each of which has a different role in establishing the dynamic of an awakening. For instance, increased dopaminergic tone results in increased theta activity, which depending on other conditions may be sufficient to induce an awakening (Vetrivelan et al., 2010). Similarly, cholinergic neurons provide significant excitability and gamma rhythms to cortical neurons (Simon et al., 2010). Serotonin neurons are not particularly efficient at eliciting sleep-to-wake transitions, but are essential gatekeepers of REM sleep (Monti, 2010a). Histamine neurons provide pacemaking signals to sleep and wake duration (Lin et al., 2011). Norepinephrine neurons in the LC have long been shown to provide diffuse excitatory input to the neocortex and efficiently promote awakenings (Carter et al., 2010, 2012). Combinatorial action of neuromodulators (e.g., increased cholinergic tone, decreased serotonin, etc) may predispose the neocortex to undergo a state transition. Hcrt thus is a powerful orchestrator of all these players in the dynamic of sleep/wake cycles.

In addition to the LC, alternative pathways such as dopaminergic, serotonergic or cholinergic systems also result in enhanced probability of arousal (**Figure 2**). Slow dynamics of neuromodulators (between 1 and 30 s) are consistent with a behavioral state transition that needs time to integrate and decide the most physiologically sensible solution. Hcrt neurons integrate multiple variables from circadian, metabolic and limbic structures. This integration is non-redundant, as Hcrt dysfunction results in uncoordinated intrusions of sleep into wakefulness associated with narcolepsy. However, other redundant integrators may exist (e.g., GABAergic systems in the lateral hypothalamus including Leptin-sensitive neurons). Information from the integrating systems is conveyed into an array of systems that have different roles in the dynamics of sleep to wake transitions. For instance, high serotonergic tone inhibits REM sleep (Monti, 2010b). Histamine neurons in the TMN fire during waking and set the length of wake bouts. Cholinergic neurons in the basal forebrain (Arrigoni et al., 2010) and dopaminergic cells provide direct innervation to the neocortex, whereas norepinephrine is a powerful arousal-promoting factor as described above. It is noteworthy that Hcrt neurons are silent during REM sleep, as it suggests that activation of Hcrt neurons is dispensable for cortical desynchronization and cholinergic excitation. Also, the fact that Hcrt stimulation suppresses REM sleep suggests several possible mechanisms: (i) direct excitation of serotoninergic neurons in the raphe; (ii) a state-dependent modulation of cholinergic activity; (iii) reciprocal excitation/inhibition of MCH neurons recently shown to be involved in REM sleep maintenance. Thus, we underscore the relevance of Hcrt neurons in coordinating arousal centers as key elements of a switchboard, not master switches as has been proposed elsewhere in the literature. Future use of optogenetic and other state-of-the art methods to interrogate combinations of neuromodulators will provide a much more detailed mechanistic description of the role of Hcrt and effectors in the modulation of sleep/wake cycles.

## Conflict of Interest Statement

The authors declare that the research was conducted in the absence of any commercial or financial relationships that could be construed as a potential conflict of interest.
